# Mesenchymal stem cell implantation provides short-term clinical improvement and satisfactory cartilage restoration in patients with knee osteoarthritis but the evidence is limited: a systematic review performed by the early-osteoarthritis group of ESSKA-European knee associates section

**DOI:** 10.1007/s00167-023-07575-w

**Published:** 2023-09-22

**Authors:** Hamid Rahmatullah Bin Abd Razak, Katia Corona, Trifon Totlis, Li Yi Tammy Chan, Jose Filipe Salreta, Obeida Sleiman, Michele Vasso, Mike H. Baums

**Affiliations:** 1 Department of Orthopaedic Surgery, Sengkang General Hospital, 110 Sengkang East Way, Singapore, 544886 Singapore; 2 Orthopedics and Traumatology, Fondazione Policlinico Universitario A. Gemelli IRCCS-Sacred Heart Catholic University, Rome, Italy; 3 Thessaloniki Minimally Invasive Surgery (The-MIS) Orthopaedic Centre, St. Luke’s Hospital, Thessaloniki, Greece; 4 Department of Anatomy and Surgical Anatomy, School of Medicine, Faculty of Health Sciences, Aristotle University of Thessaloniki, Thessaloniki, Greece; 5 Yong Loo Lin School of Medicine, National University of Singapore, 10 Medical Drive, Singapore, 117597 Singapore; 6 Orthopaedic and Traumatology Department, Hospital Garcia de Orta, Almada, Portugal; 7 Department of Orthopedics, Trauma Surgery and Sports Traumatology, Catholic Clinical Center Ruhr North (KKRN), Dorsten, Germany; 8 Department of Medicine and Health Sciences, University of Molise, Via Francesco De Sanctis, 86100 Campobasso, Italy

**Keywords:** Knee osteoarthritis, Knee OA, Mesenchymal stem cells, MSCs, Outcomes, Orthobiologics, Cartilage

## Abstract

**Purpose:**

Implantation of mesenchymal stem cells (MSCs) is a potential cell-based modality for cartilage repair. Currently, its clinical use largely surrounds focal cartilage defect repair and intra-articular injections in knee osteoarthritis. The MSCs’ implantation efficacy as a treatment option for osteoarthritis remains contentious. This systematic review aims to evaluate studies that focused on MSCs implantation in patients with knee OA to provide a summary of this treatment option outcomes.

**Methods:**

A systematic search was performed in PubMed (Medline), Scopus, Cinahl, and the Cochrane Library. Original studies investigating outcomes of MSCs implantations in patients with knee OA were included. Data on clinical outcomes using subjective scores, radiological outcomes, and second-look arthroscopy gradings were extracted.

**Results:**

Nine studies were included in this review. In all included studies, clinical outcome scores revealed significantly improved functionality and better postoperative pain scores at 2–3 years follow-up. Improved cartilage volume and quality at the lesion site was observed in five studies that included a postoperative magnetic resonance imaging assessment and studies that performed second-look arthroscopy. No major complications or tumorigenesis occurred. Outcomes were consistent in both single MSCs implantation and concurrent HTO with MSCs implantation in cases with excessive varus deformity.

**Conclusion:**

According to the available literature, MSCs implantation in patients with mild to moderate knee osteoarthritis is safe and provides short-term clinical improvement and satisfactory cartilage restoration, either as a standalone procedure or combined with HTO in cases with axial deformity. However, the evidence is limited due to the high heterogeneity among studies and the insufficient number of studies including a control group and mid-term outcomes.

**Level of evidence:**

IV.

## Introduction

Within the scope of knee joint preservation in early osteoarthritis, the hierarchy of the treatment strategy is to first optimize alignment, second achieve joint stability, followed by undergoing meniscus procedures and/or cartilage surgery [1]. High tibial osteotomy (HTO) effectively corrects alignment and improves pain and knee function in young patients with medial osteoarthritis and varus deformity [5]. However, severe articular degeneration in the affected compartment is a poor prognostic factor for HTO outcomes [3, 34]. In such cases, osteotomy can be combined with a cartilage repair technique to fill the defect [28], which may lead to improved results compared to HTO alone [7].

Mesenchymal stem cell (MSC) implantation as a potential cell-based modality for cartilage repair has had promising outcomes in clinical studies when used in patients with knee OA, either combined with HTO in patients with varus deformity [24] or as a standalone procedure in patients with no axial deformity [23, 36]. MSCs can be surgically implanted into the lesion or injected into the knee joint. Current literature has reviewed injected MSCs in patients with knee OA [6, 31], however, there is a lack of systematic reviews on surgically implanted MSCs. One systematic review evaluated outcomes of both injected and surgically implanted MSCs used in a diverse population including knee OA and chondral defects [22]. However, focal cartilage lesions are a completely different clinical entity from diffuse knee OA. There are also fundamental differences between treatment by injected versus implanted MSCs, especially regarding precise delivery of MSCs into the lesion. Therefore, a new systematic review designed with less heterogeneity in terms of diagnosis and MSCs administration technique is helpful.

To fill this literature gap, the present systematic review evaluates studies that focused on MSCs implantation in patients with knee OA providing an up-to-date summary of clinical, radiological, and second-look arthroscopy outcomes. Hypothesis is that MSCs implantation is associated with satisfactory postoperative outcomes but with contentious duration of improvement.

## Materials and methods

This systematic review was performed in accordance to the Preferred Reporting Items for Systematic reviews and Meta-Analyses (PRISMA) guidelines [21].

### Search strategy and search eligibility criteria

A comprehensive search was systematically conducted in PubMed (Medline), Scopus, CINAHL, and Cochrane Central databases. The following search algorithm was used for all databases: (“mesenchymal stem cells” OR MSCs) AND (osteoarthritis OR OA OR degeneration OR gonarthrosis) AND knee. The search was performed by two independent investigators (HR and LC) and was updated just before the final analyses on 3rd March 2023.

To be included in this review, the studies must fulfil the following predefined criteria: (i) clinical trials of any level of evidence, reporting outcomes of MSCs implantation in patients with knee OA, and (ii) studies published in English language.

The predefined exclusion criteria were: (i) studies on knee OA patients treated with injected MSCs, (ii) studies evaluating focal/isolated chondral lesions and defects, (iii) cadaveric, laboratory or animal studies and (iv) secondary research articles (e.g., systematic reviews, meta-analyses, letters to the editor or commentaries).

### Study selection

Two investigators (HR and TT) independently assessed the titles and abstracts of all identified records. The same investigators screened the full texts of all potentially eligible studies independently, according to the defined inclusion criteria. Additionally, references of the included studies were retrieved and manually reviewed to identify further eligible articles, according to the snowball method. Investigators were blinded to each other throughout the study selection and data extraction processes. Any disagreements or discrepancies were resolved by consensus.

### Quality assessment

The methodology of the study was assessed using the list of criteria as recommended by the Cochrane Collaboration and an Oxford Centre for Evidence-Based Medicine (OCEBM) level of evidence (LoE) was assigned to each study. The quality of included studies was evaluated using different scales based on study design: randomised controlled trials or non-randomised clinical studies. Randomised controlled trials were assessed by the modified Jadad scale that is an 8-point scale based on the domains: randomisation blinding, account for lost to follow-ups, eligibility criteria, adverse effects, and statistical analysis [10]. Non-randomised clinical studies were assessed by the MINORS (Methodological Index for Nonrandomized Studies) score based on the following domains: (i) clearly stated aim, (ii) inclusion of consecutive patients, (iii) prospective data collection, (iv) endpoints appropriate to the aim of the study, (v) unbiased assessment of the study endpoints, (vi) follow-up period appropriate to study aim, (vii) loss to follow-up of less than 5%, (viii) prospective calculation of the study size. If the non-randomised study was a comparative study, additional domains were assessed: (ix) adequate control group, (x) contemporary group, (xi) baseline group equivalence, and (xii) adequate statistical analysis. Each item was scored from 0 to 2 points, with a global ideal score of 16 points for non-comparative studies and of 24 points for comparative studies. Two authors (MV and KC) performed this evaluation, which included a discussion to reach a consensus in case of disagreement.

### Data extraction and outcomes

Two authors (HR and OS) independently extracted data from eligible studies using a data extraction form that was predefined according to the protocol. For each study, characteristics of participant (i.e., sample size, age, and gender), type of cartilage restorative procedure and, if present, type of control procedure, lesion size and location, grade of osteoarthritis, number of applied MSCs, follow-up, clinical and radiological outcomes as well as second-look arthroscopy findings were recorded. Clinical improvement of the patients, which was evaluated using patient reported outcome measures (PROMs), were the primary outcomes of the current study. Radiologic and arthroscopic appearance of the treatment site were secondary outcomes.

### Statistical analysis

Mean and standard deviation were calculated for continuous variables while absolute and relative frequencies were reported for categorical variables. Due to heterogeneity of the included studies in relation to study protocols, no data pooling and meta-analysis was performed.

## Results

### Study identification

**Fig. 1:**
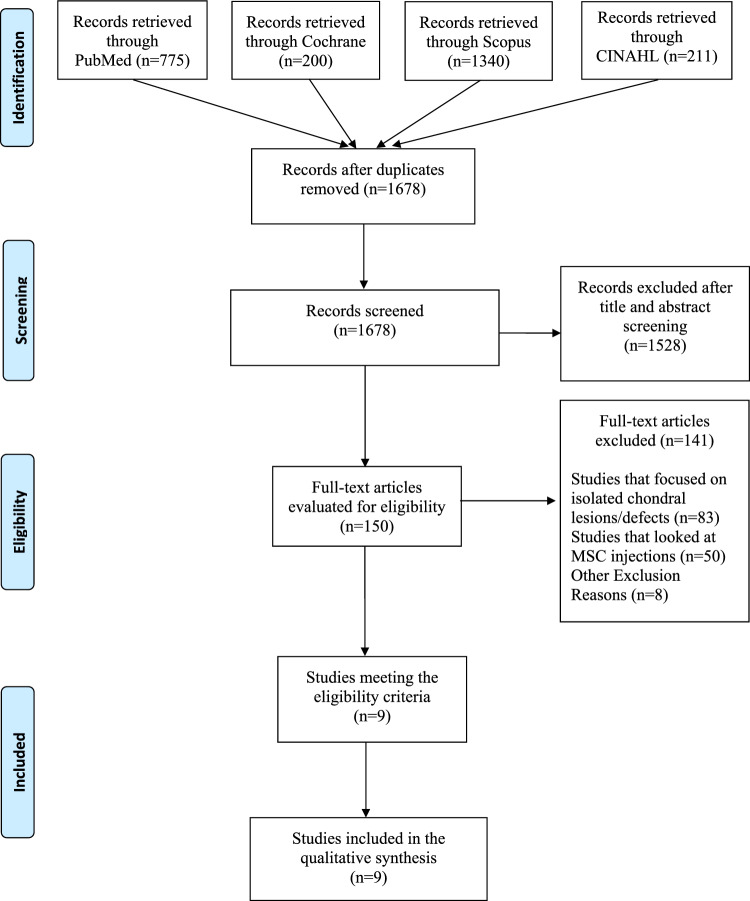
PRISMA flowchart for included studies

The PRISMA flowchart is illustrated in Fig. 1. Initially, 2526 articles were identified. After evaluation for eligibility based on inclusion and exclusion criteria nine articles finally met the inclusion criteria [15, 16, 18–20, 25, 29, 30, 36].

### Quality assessment

The mean QoE was 12.8 for non-comparative studies [15, 19, 20, 29, 30] and 18.5 for comparative studies [18, 36]. The randomised controlled trial was scored 5 by the modified Jadad scale [16, 25] (Table 1).

**Table 1:** Study design and quality assessment

Study	LoE	Country	Study design	QoE score/total
Kim et al. Am J Sports Med [18]	3	South Korea	RE	MINORS 17/24
Kim et al. Osteoarthritis Cartilage [15]	2	South Korea	PRO	MINORS 13/16
Park YB et al. Stem Cells Transl Med [25]	2	South Korea	PRO	MINORS 12/16
Kim et al. Knee Surg Sports Traumatol Arthrosc [16]	1	South Korea	RCT	MJS 5/8
Kim et al. Orthop J Sports Med [19]	4	South Korea	RE	MINORS 14/16
Song et al. Regen Ther [29]	4	South Korea	RE	MINORS 12/16
Song et al. World J Stem Cells [30]	4	South Korea	RE	MINORS 12/16
Kim et al. Orthop J Sports Med [20]	4	South Korea	RE	MINORS 14/16
Yang et al. Knee Surg Sports Traumatol Arthrosc [36]	3	South Korea	RE	MINORS 20/24

*MINORS* methodological index for non-randomised studies, *MJS* modified jadad scale, *PRO* prospective cohort study, *RCT* randomized control trial, *RE* retrospective cohort study

### Patient and study characteristics

A total of 1058 knees with knee OA were included in this review. MSCs implantation was performed in 983 knees using either autologous adipose-derived MSCs [15, 18, 19] or allogenic human umbilical cord blood-derived MSCs (hUCB-MSCs) in combination with hyaluronic acid hydrogel (Cartistem, MEDIPOST, Seongnam, Gyeonggi-do, South Korea) [25, 29]. Four studies performed MSCs implantation in association with HTO [16, 20, 30, 36]. There were 387 (36.6%) males and 671 (63.4%) females with a mean age of 57.7 years and mean postoperative follow-up period of 36.3 months (Table 2).

**Table 2:** Demographic and other characteristics of the included studies

Study	Treatment group(s)	Number of knees, N	Gender M:F (%F)	Mean age ± SD, years	Lesion size ± SD, cm^2^	Lesion location	Number of applied MSCs ± SD, × 10^6^	Pre-treatment patients’ status	Mean follow-up ± SD, months
Kim et al. [18]	MSCs implantation; MSCs injection	20; 20	14:26 (65)	59.1 ± 3.5	5.8 ± 1.8; 5.44 ± 1.4	MFC: 14; 13 LFC: 5; 6 T: 1; 1	3.96 ± 0.3; 4.07 ± 0.4	K-L 1–2 Full-thickness lesion Varus or valgus malalignment ≤ 5°	28.8 ± 4; 28.5 ± 4.8
Kim et al. [15]	MSCs implantation	20	11:9 (45)	57.9 ± 5.9	5.8 ± 1.8	MFC: 14 LFC: 5 T:1	3.96 ± 0.3	K-L 1–2 Varus or valgus malalignment < 5°	27.9 ± 3.2
Park YB et al. [25]	Allogeneic hUCB-MSCs implantation with HA	7	2:5 (71)	58.7 ± 8.9	5.9 ± 1.4	MFC: 5 LFC: 2	14.7 ± 1.6	K-L 3 ICRS grade 4 lesion > 2 cm^2^	84
Kim et al. [16]	MSCs implantation with HTO; MSCs implantation with AC with HTO	36; 34	29:41 (59)	55.6 ± 2.9; 56.1 ± 3.6	FC: 5.6 ± *2*.6; 5.6 ± 2.4 TP: 4.5 ± 1.6; 4.6 ± 1.5	NR	4.7	K-L 3–4 Varus (angle NR) malalignment	27.3 ± 3.3; 27.8 ± 3.9
Kim et al. [19]	MSCs implantation	483	150:333 (69)	61.1 ± 6.6	MFC:7 ± 1 TP:6.1 ± 0.9 T:5.4 ± 0.4	MFC:320 LFC:148 T:15	8.11	K-L 1–2 Full-thickness lesion Varus or valgus malalignment ≤ 5°	86.3 ± 13.7
Song et al. [29]	Allogeneic hUCB-MSCs implantation	128	86:42 (33)	56.5 ± 7.9	MFC:4.3 ± 1.2 LFC:5.2 ± 1 T:4.6 ± 1.6	MFC:38 LFC:6 T:22	7.5	K-L 1–3 Full-thickness lesion ≥ 2 cm^2^ Varus or valgus malalignment < 8°	36.1 ± 6.4
Song et al. [30]	Allogeneic hUCB-MSCs implantation with HTO	125	30:95 (76)	58.3 ± 6.8	6.9 ± 2	T:73	7.5	K-L 1–3 Full-thickness lesion > 4 cm^2^ Varus malalignment > 5°	36.0
Kim et al. [20]	MSCs implantation with HTO	75	35:40 (53)	60.2 ± 6.1	FC: 7.1 ± 1 TP: 6.2 ± 0.9	NR	11.9 ± 3.7	K-L 3–4 Varus (angle NR) malalignment	26.8 ± 3.1
Yang et al. 2022 [36]	Allogenic hUCB-MSCs implantation with HTO; HTO with BMAC	55; 55	30:80 (73)	56.4 ± 5.3; 55 ± 7.3	6.2 ± 2.4; 6.4 ± 3.1	NR	7.5	K-L 3–4 Lesion grade ≥ ICRS grade 3 Varus (angle NR) malalignment	31.0 ± 6.0; 34.2 ± 8.4

*AC* allogenic cartilage, *BMAC* bone marrow aspirate concentrate, *FC* femoral condyle, *HA* hyaluronic acid, *HTO* high tibial osteotomy, *hUCB-MSCs* human umbilical cord blood-derived mesenchymal stromal cells, *K-L* Kellgren-Lawrence, *LFC* lateral femoral condyle, *MFC* medial femoral condyle, *MSCs* mesenchymal stromal cells, *NR* not reported, *OA* osteoarthritis, *T* trochlea, *TP* tibial plateau, *ICRS* international cartilage repair society

### Clinical outcomes

The International Knee Documentation Committee subjective score (IKDC) [27] was used by seven studies. There was a mean improvement from 31.6 preoperatively to 64.8 at final follow-up [15, 18, 19, 25, 29, 30, 36]. The Tegner Activity score was used in four studies [15, 18, 19, 36] reporting an average improvement from 2.3 preoperatively to 3.8 postoperatively. Overall, clinical outcome scores reported by the included studies significantly improved after MSCs implantation (Table 3).

**Table 3:** Clinical outcomes across included studies

Study	Treatment	IKDC Score			Tegner activity Scale			KOOS			VAS	Others	Reported correlations
		Preoperatively	Second-look arthroscopy	Final follow-up	Preoperatively	Second-look arthroscopy	Final follow-up	Preoperatively	Second-look arthroscopy	Final follow-up			
Kim et al. Am J Sports Med [18]	MSCs implantation; MSCs injection	38.5 ± 9.2; 36.6 ± 4.9	55.2 ± 15.0; 62.7 ± 14.1	55.8 ± 14.7; 64.8 ± 13.4	2.5 ± 1.2; 2.3 ± 0.9	3.5 ± 1.2; 3.6 ± 1.1	3.5 ± 1.0; 3.9 ± 1.0	NR	NR	NR	NR	NA	Overall, implantation group had significantly better mean IKDC scores at final follow-up than injection group (*P* = .049)
Kim et al. Osteoarthritis Cartilage [15]	MSCs implantation	38.7 ± 7.0	NR	67.3 ± 11.6	2.5 ± 0.9	NR	3.9 ± 0.7	NR	NR	NR	NR	NA	As quality of repaired cartilage increased, IKDC and tegner activity score increased (*P* < 0.05 for all)
Park YB et al. Stem Cells Transl Med [25]	Allogeneic hUCB-MSCs implantation with HA	39.1	NR	63.2	NR	NR	NR	NR	NR	NR	Preop 49.1 Final follow-up 19.3	NA	NA
Kim et al. Knee Surg Sports Traumatol Arthrosc [16]	MSCs implantation with HTO; MSCs implantation with AC with HTO	NR	NR	NR	NR	NR	NR	42.5 ± 16.5; 41.7 ± 15.7	63.2 ± 14.3; 65.3 ± 14.9	67.3 ± 17.2; 73.6 ± 17.8	NR	Lysholm preop 57.8 ± 11.9; 58.6 ± 12.1 Second-look arthroscopy 81.2 ± 14.9; 84.9 ± 15.2 Final follow-up 85.4 ± 15.9; 89.3 ± 16.1	Mean KOOS scores improved significantly at the time of second-look arthroscopy in both groups (*P* < 0.001) compared to preoperative values At final FU, mean KOOS scores were further improved in MSC-AC group (*P* < 0.05) but not in MSC group
Kim et al. Orthop J Sports Med [19]	MSCs implantation	39.2 ± 7.2	NR	62.8 ± 8.5	2.3 ± 1.0	NR	3.2 ± 0.9	NR	NR	NR	NR	NA	NA
Song et al. Regen Ther [29]	Allogeneic hUCB-MSCs implantation	32.5 ± 8.3	NR	61.2 ± 17.2	NR	NR	NR	NR	NR	NR	Preop 7.0 ± 1.6 Final follow-up 2.0 ± 2.1	WOMAC preop 39.3 ± 12.2 Final follow-up 13.9 ± 14.1	Preoperatively, there were no significant differences in IKDC, WOMAC and VAS scores among different ICRS grading groups. Postoperatively, IKDC, WOMAC and VAS scores in ICRS grade I group improved more than that of grade II and III except for IKDC at 1 year FU. IKDC scores did not differ significantly between ICRS grade II and III
Song et al. World J Stem Cells [30]	Allogeneic hUCB-MSCs implantation with HTO	32.5 ± 8.3	NR	61.2 ± 17.2	NR	NR	NR	NR	NR	NR	Preop 7.0 ± 1.6 Final follow-up 2.0 ± 2.1	WOMAC preop 39.3 ± 12.2 Final follow-up 13.9 ± 14.1	NA
Kim et al. Orthop J Sports Med [20]	MSCs implantation with HTO	NR	NR	NR	NR	NR	NR	55.1 ± 3.7	NR	82.9 ± 3.8	NR	NR	Mean KOOS scores improved significantly at final follow-up (*P* < 0.001 for all)
Yang et al. Knee Surg Sports Traumatol Arthrosc [36]	Allogenic hUCB-MSCs implantation with HTO; HTO with BMAC	35.4 ± 5.5	NR	73.3 ± 9.8	2.2 ± 0.8	NR	4.1 ± 0.5	39.5 ± 6.9	NR	79.4 ± 8.8	NR	SF-36 physical component preop 41.5 ± 5.5 Final follow-up 65.4 ± 7.9	As ICRS CRA scores increased (quality of cartilage regeneration increased), IKDC scores increased significantly (*r* = − 0.337, *P* = 0.002)

*ICRS* international cartilage repair society, *IKDC* international knee documentation committee subjective knee form, *KOOS* knee injury and osteoarthritis outcome score, *MSC* mesenchymal stem cell, *MSC-AC* mesenchymal stem cell and allogenic cartilage, *NA* not applicable, *NR* not reported, *SF-36* 36-item short form survey, *WOMAC* western ontario and mcmaster universities osteoarthritis index

### Imaging outcomes

Three studies evaluated the quality of repaired cartilage with the Magnetic Resonance Observation of Cartilage Repair tissue (MOCART) scoring system [15, 20, 29] and reported an average score of 68.3 points at the treatment site. Another study found high glycosaminoglycan content of the regenerated cartilage (ΔR1 index 1.44) post-transplantation, using delayed gadolinium-enhanced MRI of the cartilage (dGEMRIC) (Table 4) [25].

**Table 4:** Key findings from radiological outcomes among included studies

Study	Plain radiographs	MRI	
		MOCART score (Follow-up time in months)	Others
Kim et al. Osteoarthritis Cartilage [15]	NR	69.8 ± 14.3 (24.2)	Cartilage lesion grades described by MOAKS at follow-up were significantly better than preoperative values (*P* < 0.001)
Park YB et al. Stem Cells Transl Med [25]	NR	NR	A delayed gadolinium-enhanced MRI of the cartilage was performed at 3 years follow-up and results indicated high glycosaminoglycan content of the regenerated cartilage (ΔR1 index 1.44)
Kim et al. Knee Surg Sports Traumatol Arthrosc [16]	Femorotibial angle (MSC; MSC-AC) Preop: varus 3.2° ± 1.9 to varus 3.2° ± 1.8 Final follow-up: valgus 8.9° ± 2.8; valgus 8.8° ± 2.7 Posterior tibial slope (MSC; MSC-AC) Preop 10.3° ± 3.6; 10.2° ± 3.2 Final follow-up 10.5° ± 2.8; 10.4° ± 2.7	NR	NA
Kim et al. Orthop J Sports Med [19]	Gradual deterioration of radiological outcomes according to the KL grade was found after a follow-up period of 5 years	NR	NA
Song et al. Regen Ther [29]	NR	30.58 (3.8) to 55.44 (21.2)	NA
Kim et al. Orthop J Sports Med [20]	NR	Femoral condyle 74.2 ± 8.6 (26.8) Tibial condyle 74.1 ± 7.5 (26.8)	NA
Yang et al. Knee Surg Sports Traumatol Arthrosc [36]	Improved knee joint alignment after MSC implantation Posterior tibial slope from 7.9° ± 2.1 to 8.2° ± 2.5	NR	NA

*MOAKS* MRI osteoarthritis knee score, *MOCART* magnetic resonance observation of cartilage repair tissue, *MSC* mesenchymal stem cells, *MSC-AC* mesenchymal stem cells with allogenic cartilage, *NA* not applicable, *NR* not reported

### Second-look arthroscopy outcomes and survival rate

Among the five studies that performed second-look arthroscopy, three observed an improvement to the cartilage status according to the ICRS grading system [18, 30, 36] and two reported cartilage regeneration using other evaluation tools (Table 5) [16, 25]. One study reported survival rates based on either a decrease in IKDC or an advancement of radiographic OA with K-L scores. An IKDC score below 40 or deterioration of radiologic outcomes from K-L grade 1 or 2 to K-L grade 3 or 4 was defined as failure. According to this definition, the reported survival rates were 99.8%, 94.5%, and 74.5% at 5, 7, and 9 years, respectively [19]. In another case series, no significant deterioration of VAS and IKDC scores was observed 7 years postoperatively [25].

**Table 5:** Cartilage outcomes at second-look arthroscopy

Study	Outcomes at second-look arthroscopy	
	MSC implantation group	Control group
Kim et al. Am J Sports Med [18]	ICRS grading: Grade I: 6/20 (30%) Grade II: 7/20 (35%) Grade III: 4/20 (20%) Grade IV: 3/20 (15%) The ICRS grades were significantly better in the MSCs implantation group (P = .041)	ICRS grading: Grade I: 2/20 (10%) Grade II: 5/20 (25%) Grade III: 8/20 (40%) Grade IV: 5/20 (25%)
Park YB et al. Stem Cells Transl Med [25]	The arthroscopic examination at 1 year revealed good resurfacing with thick and glossy white hyaline-like cartilage at the lesion site. The regenerated cartilage had a smooth surface with firm consistency and showed good integration with the surrounding native cartilage	NR
Kim et al. Knee Surg Sports Traumatol Arthrosc [16]	Kanamiya grading in MSC implantation only without allogenic cartilage: – 38.9% of lesions were grade 3 or 4 on the femoral condyle – 38.9% were grade 3 or 4 on the tibial plateau	Kanamiya grading in MSC implantation with allogenic cartilage: – 58.9% of lesions in were grade 3 or 4 on the femoral condyle – 55.9% of lesions were grade 3 or 4 on the tibial plateau The overall Kanamiya grades were better in the knees which underwent MSC implantation with allogenic cartilage
Song et al. World J Stem Cells [30]	ICRS grading of medial femoral condyle cartilage: Grade I: 73/125 (58.4%) Grade II: 37/125 (29.6%) Grade III: 15/125 (12%) Grade IV: 0	NR
Yang et al. Knee Surg Sports Traumatol Arthrosc [36]	ICRS cartilage repair assessment scoring in knees which underwent MSC implantation: 9.2 ± 2.2	ICRS cartilage repair assessment scoring in knees which underwent BMAC: 7.2 ± 3.0

*BMAC* bone marrow aspiration concentrate, *ICRS* international cartilage repair society, *MSC* mesenchymal stem cell, *NR* not reported

## Discussion

The most important finding of this systematic review is that in all included studies clinical outcome scores revealed significantly improved functionality and better postoperative pain scores in patients with knee OA who underwent MSCs implantation at 2–3 years follow-up. Improved cartilage volume and quality at postoperative MRI and second-look arthroscopy was consistently observed. No major complications or tumorigenesis occurred. Outcomes were consistent in both single MSCs implantation and concurrent HTO with MSCs implantation in cases with excessive varus deformity.

There is no current consensus on the MSCs optimal therapeutic dose to be implanted for cartilage regeneration as demonstrated by the range of MSCs concentrations used among eligible studies in the present systematic review (3.96–11.9 × 10^6^ cells). To add to the ambiguity, the estimation of MSCs dosage may vary as some studies included the entire mononuclear cell count in dosage calculation, involving heterogenous population of cells such as platelet-rich plasma (PRP), autologous conditioned serum (ACS) apart from MSCs. Nonetheless, the current study suggests that clinical outcomes after MSCs’ implantation are significantly influenced by MSCs’ counts [18–20]. These findings concur with a recent meta-analysis, which found incremental improvement in VAS and KOOS with increasing dosage of MSCs injections at 12 months [23]. Further studies of sufficient power and duration should be carried out to arrive at a definitive consensus on the prevailing ambiguity in the volume and count of MSCs needed in MSCs-based treatment of knee OA. Apart from the number of MSCs, patient age and presence of bipolar kissing lesion were also independent factors associated with failure of MSCs’ implantation [23, 24].

Delaying total knee arthroplasty (TKA) is important because the incidence of primary TKA is increasing among younger age patients. There is evidence that the risk of revision TKA and dissatisfaction increases as the age of the patient decreases [14]. While this study provides encouraging evidence that utilizing MSCs can control symptoms and improve function and cartilage volume at the lesion site, there is no evidence about the long-term efficacy after MSCs implantation or injection. Kim et al. observed significant deterioration in clinical outcome scores after 3 years and OA K-L grade after 5 years [19], whereas Park et al. found no significant deterioration of VAS and IKDC scores at 7 years follow-up [25]. Another study by Hernigou et al. showed that the benefits of MSCs intra-articular and/or subchondral injection may last up to 15 years in some knees [8]. During the 15-years’ follow-up period, they found that 20% of patients converted to subsequent TKA after MSCs implantation, with an incidence of 1.3% per year.

Among the included studies, all methods have shown benefit in clinical outcomes despite the heterogeneity in lesion location, number of cells, grading of OA and follow-up periods. There are reports of cartilage status improvement in the affected compartment after HTO without any concomitant cartilage procedures [11]. However, there are no studies to date comparing outcomes following HTO alone versus HTO with MSCs implantation in OA. Combined MSCs’ injection and HTO has been associated with significantly better clinical scores than HTO alone in patients with knee OA [17]. In the systematic review by Kahlenberg et al., results of the second-look arthroscopy were mixed, with two studies showing significant improvement in the cartilage with HTO plus cartilage restoration procedures versus HTO alone, whereas another study showed no difference [13]. Recently, Bode et al. demonstrated a 87.2% 10-years survival rate for HTO alone and 94.3% for the HTO plus autologous chondrocyte implantation subgroup [2]. Larger scale comparative studies are needed for cartilage restoration techniques to determine whether they have a significant impact on fibrocartilage growth, clinical outcomes and TKA delay after HTO.

The present study revealed that radiological outcomes based on MRI and second-look arthroscopy correlate with clinical outcomes. However, plain radiographic outcomes were not significantly correlated with clinical outcomes [16, 20]. A potential explanation is that cartilage regeneration following MSCs implantation is not adequate to induce an improvement in OA staging on plain radiographs. This may suggest the need for clinicians to perform postoperative cartilage-sequence MRIs in addition to plain radiographs to evaluate quality of repaired cartilage.

The findings of this study must be interpreted in light of its limitations. Firstly, heterogeneity in terms of MSCs source and primary outcomes reported in the studies may have affected the analysis and data interpretation. Secondly, several included studies in this review were from South Korea, and from the same principal investigator which adds to bias [15, 16, 18–20]. Thirdly, confounding effects may be present due to concurrent treatment (HTO) in four out of nine eligible studies. However, this best represents the current clinical practice in which knee preservation is performed. Next, most of the included studies had a follow-up period of two to three years [15, 16, 18, 20, 25, 29, 30, 36]. To make a firmer and safer conclusion regarding the efficacy of MSCs implantation along with the optimal patient selection, more studies with diversified cohort and longer follow-up period need to be conducted. Lastly, there was little information among included studies regarding pre-treatment cartilage status of the patients. Therefore, no meaningful comparison could be made across study groups pre- and post-treatment with MSCs’ implantation.

MSCs have been suggested for treatment of knee OA since their differentiation into chondrocytes can lead to cartilage repair. Next, homing characteristics of MSCs make them ideal seed cells for gradual OA treatment [1, 4, 33]. In systemic homing, MSCs administered into the bloodstream may undergo a multistep process to exit the circulation and migrate to the site of injury such as the knee to modify the disease. Considering the pathogenesis of OA, the paracrine [1, 9], anti-inflammatory [32], and immunomodulatory [12, 35] effects of MSCs may provide additional benefit by improving the intra-articular environment aiming to modify OA disease progression [26]. However, all these MSCs’ properties and capabilities are on a theoretical basis. The present systematic review summarized the short-term clinical, radiological and second-look arthroscopy outcomes to provide the current evidence on MSCs implantation potential use as a joint-preserving treatment, either as a single procedure or combined with HTO in cases with axial deformity, especially in young patients with knee OA who aim to avoid or delay arthroplasty.

## Conclusion

According to the available literature, MSCs’ implantation in patients with mild to moderate knee osteoarthritis is safe and provides short-term clinical improvement and satisfactory cartilage restoration, either as a standalone procedure or combined with HTO in cases with axial deformity. However, the evidence is limited due to the high heterogeneity among studies and the insufficient number of studies including a control group and mid-term outcomes.

## Data Availability

Not applicable.

## Abbreviations

MSCs: Mesenchymal Stem Cells
OA: Osteoarthritis
PRISMA: Preferred reporting items for systematic reviews and meta-analyses
OCEBM: Oxford centre for evidence-based medicine
LoE: Level of evidence
MINORS: Methodological index for nonrandomized studies
hUCB-MSCs: Human umbilical cord blood-derived MSCs
HTO: High tibial osteotomy
IKDC: International knee documentation committee
KOOS: Knee injury and osteoarthritis outcome score
VAS: Visual analogue scale
MRI: Magnetic resonance imaging
K-L: Kellgren–Lawrence
MOCART: Magnetic resonance observation of cartilage repair tissue
MOAKS: MRI osteoarthritis knee score
ICRS: International cartilage repair score
